# Newborn gender as a predictor of neonatal outcome in mixed gender twins born with very low birth weight

**DOI:** 10.1186/s12887-019-1713-2

**Published:** 2019-09-11

**Authors:** Bernard Barzilay, Nina Shirman, Haim Bibi, Ibrahim Abu-Kishk

**Affiliations:** 1 Neonatal Intensive Care Unit, Shamir Medical Center (Assaf Harofeh), Zerifin, affiliated to the Sackler Faculty of Medicine, Tel-Aviv University, Tel-Aviv, Israel; 2 Pediatric Intensive Care Unit, Shamir Medical Center (Assaf Harofeh), Zerifin, affiliated to the Sackler Faculty of Medicine, Tel-Aviv University, Tel-Aviv, Israel; 3 Pediatric Division, Shamir Medical Center (Assaf Harofeh), 7033001 Zerifin, Israel

**Keywords:** Twins, Gender, Very low birth weight, Neonatal morbidity, Neonatal mortality

## Abstract

**Background:**

Most studies have revealed that the incidence of morbidity and mortality of preterm male infants is greater than that of preterm female infants. Recently, conflicting outcomes have been reported regarding mixed-gender twins. The aim of this study was to estimate the association between gender and outcome in newborn twins of different gender.

**Methods:**

We conducted a retrospective review of mixed-gender twins weighing < 1500 g that were born at Shamir Medical Center (Assaf Harofeh) between the years 1995 and 2016 (158 newborns). The incidence of morbidity and mortality until discharge from the hospital were evaluated while looking at gender differences.

**Results:**

No significant differences were found in neonatal mortality or morbidity between females and males from different-gender twins. Even after considering confounding variables (gestational age, birth weight & birth order) in linear and logistic regression models, no significant differences were found between the genders.

**Conclusions:**

Our study suggests that there are no significant differences in neonatal mortality or morbidity among different-gender twins. Our results support the need for further studies.

## Background

Prematurity and low birth weight is a major risk factor for neonatal morbidity and mortality [1]. Premature birth is more common among male infants than female infants [2], and among twins, there is a higher incidence of premature births of male-male premature twins than female-female or female-male twins [3, 4]. Furthermore, mortality rates and short-term morbidity are higher among male premature infants than females [5–9].

Studies revealed that the prognosis of males who are part of a male-male pair relative to a female-female pair is less favorable [10–12]. This difference is particularly prominent mainly in regard to respiratory morbidity, especially respiratory distress syndrome (RDS) [11, 13]. Shinwell et al. [10] showed that male/male twins were at increased risk for death, RDS, pneumothorax, bronchopulmonary dysplasia (BPD), periventricular hemorrhage (PVH) and periventricular leukomalacia (PVL) [10].

Recently two studies were published on comparisons between males and females in cases of mixed gender twins. Zhao et al. [14] showed that males had an increased risk of mortality, lower Apgar score and higher incidence of RDS. However, Ahrenfeldt et al. [12] presented conflicting results, with no differences in mortality rates between males and females [12].

Due to the conflicting data, we conducted a study based on our population. Since very low birth weight premature neonates are the most vulnerable [15], we chose to examine this population subgroup in order to better reflect the gender differences regarding the outcome.

## Methods

We conducted a retrospective review of all the premature mixed gender twins that were born at Shamir Medical Center (Assaf Harofeh) between the years 1995 and 2016 (158 newborns). Inclusion criteria: birth weight of less than 1500 g. Exclusion criteria: twins who were born dead and babies with severe structural and/or chromosomal abnormalities. Details of the perinatal and post-natal course until the discharge date or death were recorded. Medical data in regard to morbidity and mortality was collected. A comparative evaluation was done between the male and female groups. The study was approved by the Institutional Ethics Committee at Shamir Medical Center (Assaf Harofeh) (Ref: 0073–17-ASF).

In each group, complications of prematurity and post-neonatal complications were examined until discharge from the neonatal intensive care unit. The data includes: delivery room information (birth weight, birth weight adjustment for gestational age, Apgar scores, neonatal status of the newborn, umbilical cord pH, resuscitation in the delivery room); and Neonatal Intensive Care Unit information [early and late sepsis, period of oxygen enrichment required, surfactant use, length of mechanical ventilation, length of hospitalization stay (LOS), RDS, necrotizing enterocolitis (NEC), periventricular leukomalacia (PVL), periventricular/intraventricular hemorrhage (PVH/IVH), bronchopulmonary dysplasia (BPD), retinopathy of prematurity (ROP) and mortality].

Since the comparison was made between the twins, it was possible to overcome confounding factors related to genetics, exposures during pregnancy and birth, and social and demographic factors.

### Clarifications

Weight Z score was presented by Fenton growth charts. PH levels refer to the umbilical cord blood pH levels. The need for resuscitation refers to the need for resuscitation in the delivery room. Invasive ventilation refers to mechanical ventilation via an endotracheal tube. Noninvasive ventilation refers to continuous positive airway pressure (CPAP) induced through a mask. Early/late onset of sepsis refers to sepsis presenting before/after the first 72 h of life. Outcome score is a calculated score between 0 and 6 and based on six parameters (mortality, BPD, NEC, ROP stages 2–4, PVH/IVH grades 3–4, PVL) with a weighted value of 0–1 [16].

### Statistical analysis

Categorical variables were described using frequency and percentage, and sequential variables were described using mean and standard deviation. Continuous variables were calculated using a t-test. The difference between the two groups dependent on categorical variables was performed using the McNemar’s test. The difference between the two groups dependent on ordinal variables was performed using the Wilcoxon test. For dichotomous variables, data was analyzed using mixed model logistic regression, which took into account the dependence between twins in order to predict the probability of mortality and complications. For sequential variables, data were analyzed using mixed linear models that took into account the dependence of the twins, and the explanatory variables were the subjects’ sex, the birth week, the Z score of the twin weights, and birth order. Data analysis was performed with the statistical software IBM SPSS statistics 24. The value of *P* < 0.05 was considered as statistically significant.

## Results

Data on 162 premature mixed gender twins was collected, from them 2 twin pairs were excluded, due to death at birth and chromosomal abnormalities. There were 158 twins included in the final study, divided into 2 equal groups (males and females). The characteristics of the participants are illustrated in Table 1. Among 77 pairs out of the total number of 79 pairs, the delivery method was the same, whereas in 2 pairs the delivery method was different (cesarean versus vaginal delivery). Regarding the birth weight, 87.3% of males and 82.3% of females were in the appropriate for gestational age group while 12.7% of males and 17.7% of females were in the small for gestational age group. In order to take into account the week of birth and the newborn’s gender with respect to weight, Z scores were calculated for neonatal weights using Fenton growth charts.

**Table 1:** Characteristics of the participants

Characteristics	Male (*N* = 79)		Female (*N* = 79)		*p*-value
	Mean	SD	Mean	SD	
Birth weight (grams)	1141.1	284.6	1076.8	275.8	0.015
Weight Z score	−0.195	0.825	−0.144	0.864	0.571
Apgar 1 min	7.4	2.1	7.3	2.1	0.702
Apgar 5 min	8.7	1.7	8.8	1.6	0.712
PH	7.17	0.98	7.3	0.7	0.311
	N (total = 79)	%	N (total = 79)	%	
Vaginal delivery	12	15.2	14	17.7	0.500^a^
Cesarean delivery	67	84.8	65	82.3	
First born	44	55.7	35	44.3	0.368
SGA	10	12.7	14	17.7	0.503^b^
AGA	69	87.3	65	82.3	
LGA	0	0	0	0	
Resuscitation	51	64.6	65	70.9	0.267

Weight Z score is according to Fenton growth charts. PH refers to levels in the umbilical cord. Birth weight is in grams corrected for gestational age. Resuscitation refers to neonatal resuscitation in the delivery room. ^a^Refers to mode of delivery (vaginal and cesarean together). ^b^Refers to SGA, AGA and LGA together. *AGA* appropriate for gestational age, *LGA* large for gestational age, *SD* standard deviation, *SGA* small for gestational age

Table 2 describes the prognostic variables of neonates. No statistical significance was found between the groups.

**Table 2:** Outcomes: Comparison between the Groups

Characteristics	Male		Female		*p*-value
	Mean (days)	SD	Mean (days)	SD	
LOS	57.3	34.3	54.1	30.3	0.428
Total ventilation	18.9	26.5	15.9	21.9	0.246
Invasive ventilation	9.7	18.7	8.6	16	0.578
O2 enrichment	25.6	31.3	23.8	27.3	0.585
	Total	N (%)	Total	N (%)	*p* value
SRT	79	43 (54.4)	79	43 (54.4)	1.000
RDS	78	52 (66.7)	78	52 (66.7)	1.000
BPD	78	17 (21.8)	78	15 (19.2)	0.744
NEC	78	2 (2.6)	78	1 (1.3)	0.344
ROP	69	13 (18.8)	66	9 (13.6)	0.625
PVH/IVH	73	5 (6.8)	72	5 (6.9)	1.000
PVL	58	2 (3.4)	60	3 (5)	1.000
Early onset sepsis	78	3 (3.8)	78	0 (0)	0.250
Late onset sepsis	75	4 (5.3)	74	7 (9.5)	0.344
Mortality	79	9 (11.4)	79	11 (13.9)	0.727
	Mean	SD	Mean	SD	p value
Outcome score	0.6	0.8	0.5	0.8	0.394

Total ventilation (days) includes nasal CPAP and invasive ventilation. Early onset sepsis refers to sepsis presenting in the first 72 h of life. Late onset sepsis refers to sepsis presenting after 72 h of life. Outcome score is a calculated score between 0 and 6, based on six parameters (mortality, BPD, NEC, ROP stages 2–4, PVH/IVH grades 3–4, PVL) with a weighted value of 0–1. *BPD* bronchopulmonary dysplasia, *CPAP* continuous positive airway pressure, *LOS* length of stay (hospitalization days), *NEC* – necrotizing enterocolitis, *PVH/IVH* periventricular/intraventricular hemorrhage grade 3–4, *PVL* periventricular leukomalacia, *RDS* respiratory distress syndrome, *ROP* retinopathy of prematurity stages 2–4, *SD* standard deviation, *SRT* surfactant replacement therapy

Multivariate analysis, using a mixed regression model, took into account the dependence between the twins in order to predict the risk of death and complications such as RDS, BPD, ROP, PVH/IVH, NEC, and PVL. The explanatory variables were the gender of the subjects, gestational age, the Z score of the twin weights, and the birth order. A significant association was found between the Z scores of birth weight and ROP; for each unit increase in Z score there was a 0.425 reduction in risk for ROP (*P* < 0.015, CI 0.214–0.845). No significant relationship was found between the newborn’s gender and the specific complications.

In addition, an analysis was performed for continuous variables using mixed linear models, which took into account the dependence of the twins in order to predict the number of days of hospitalization, number of days of oxygen supplement, number of days of ventilator support, number of days of intrusive ventilator support, Apgar score after 5 min and pH in the umbilical cord. The explanatory variables included in the model were the subjects’ sex, the birth week, Z score of the twin weights and the order of birth. No significant relationships were found between the gender of the newborn and the specific variables. A significant correlation was found between the gestational age and all the variables examined, except for pH in the umbilical cord. For each additional week of pregnancy there was a decrease in the number of hospitalization days, ventilator support (invasive and non-invasive) and length of oxygen supplement treatment (Table 3). In a mixed model logistic regression the main outcomes were associated with lower gestational age. The only other association was found between lower birth weight and NEC (Table 4).

**Table 3:** Linear Mixed Model – Clinical Characteristics and Outcome

Variable	Male	Female	*p*-value
LOS (days)	Gender	3.31	0.421
	GA	−5.16	0.000
	Z score	−6.12	0.079
	Order	2.08	0.616
O2 enrichment	Gender	2.08	0.536
	GA	−6.01	0.000
	Z score	−1.76	0.529
	Order	3.10	0.364
Invasive ventilation (days)	Gender	2.67	0.224
	GA	−5.03	0.000
	Z score	−0.21	0.928
	Order	1.84	0.498
Total ventilation (days)	Gender	1.22	0.531
	GA	−3.37	0.000
	Z score	−1.05	0.518
	Order	1.80	0.363
Apgar 1 min	Gender	0.06	0.756
	GA	0.49	0.000
	Z score	0.10	0.600
	Order	−0.26	0.197
Apgar 5 min	Gender	−0.03	0.746
	GA	0.34	0.000
	Z score	0.21	0.080
	Order	−0.08	0.455
PH	Gender	−0.17	0.204
	GA	0.04	0.218
	Z score	−0.10	0.257
	Order	−0.20	0.13

Estimate – the amount of change in the dependent variable for each increase of one unit in the independent variable. Estimate regarding gender – male versus female. Estimate regarding order – first born versus second born. Total ventilation (days) includes nasal CPAP and invasive ventilation. PH refers to levels in the umbilical cord

*CPAP* continuous positive airway pressure*, GA* gestational age, *LOS* length of stay (hospitalization days)

**Table 4:** Association between Variables and Outcomes^a^

				95% Confidence Interval of the Difference	
Variable	Parameter	Significance	Exp (Coefficient)	Lower	Upper
Mortality	Gender	0.630	0.721	0.188	2.757
	GA	**0.020**	0.478	0.257	0.887
	Weight	0.507	0.998	0.993	1.003
	Order	0.965	1.030	0.269	3.941
RDS	Gender	0.819	0.935	0.526	1.663
	GA	**0.000**	0.510	0.376	0.692
	Weight	0.284	1.001	0.999	1.003
	Order	0.858	0.949	0.534	1.687
BPD	Gender	0.364	1.329	0.717	2.465
	GA	0.105	0.799	0.609	1.048
	Weight	0.059	0.998	0.996	1.000
	Order	0.784	1.090	0.587	2.024
NEC	Gender	0.178	3.566	0.558	22.790
	GA	0.283	1.240	0.836	1.839
	Weight	**0.003**	0.992	0.988	0.997
	Order	0.912	1.117	0.157	7.939
ROP	Gender	0.194	1.582	0.789	3.171
	GA	**0.000**	0.364	0.211	0.600
	Weight	0.101	0.997	0.995	1.000
	Order	0.707	0.873	0.430	1.776
PVH/IVH	Gender	1.000	1.000	0.207	4.838
	GA	0.474	0.474	0.205	1.096
	Weight	1.000	1.000	0.994	1.007
	Order	1.670	1.670	0.352	7.920
PVL	Gender	0.632	0.635	0.098	4.125
	GA	0.732	0.895	0.471	1.700
	Weight	0.762	0.999	0.994	1.004
	Order	0.550	1.764	0.270	11.513
Resuscitation in delivery room	Gender	0.165	0.709	0.436	1.153
	GA	**0.007**	0.614	0.431	0.875
	Weight	0.736	1.000	0.998	1.001
	Order	0.960	1.012	0.626	1.637

^a^Analyzed by a mixed model for logistic regression

Boldface- *P* value <0.05, considered significant

*BPD* bronchopulmonary dysplasia, *NEC* necrotizing enterocolitis, *PVH/IVH* periventricular/intraventricular hemorrhage grade 3–4, *PVL* periventricular leukomalacia, *RDS* respiratory distress syndrome, *ROP* retinopathy of prematurity stages 2–4, *RDS* respiratory distress syndrome, *GA* gestational age

## Discussion

Our study suggests that there are no significant differences in neonatal and post neonatal mortality and morbidity among different gender preterm twins with birth weight less than 1500 g. Birth weight was found to be significantly higher in males; however, in the calculation of Z score for weight depending on gender and gestational week at birth, there was no significant difference between males and females. In the logistic regression model, a significant association was found between the Z score of the weight and ROP. For each increase in standard deviation in the Z score, the odds of ROP decreased. In addition, a significant correlation was found between the week of birth and most of the prognosis variables examined (including mortality, RDS, BPD, ROP, LOS, length of mechanical ventilation and oxygen supplement duration); with each additional gestational week at birth reducing the risk of the detailed prognosis variables and increasing the Apgar score at 1 and 5 min.

Zhao et al. [14] reported that males had an increased risk of mortality, lower Apgar score and higher incidence of RDS. Their data and review of the literature did not reveal the exact mechanism for this phenomenon, and the exact process that affects the gender related difference in morbidity and mortality is still unknown. Zhao et al. studied a large number of neonates from all ranges of birth weight and gestational age. We chose only very low birth weight neonates since this population is the most vulnerable and sensitive in term of morbidity and mortality [15]. From our point of view, this highly sensitive population may reflect better the differences between genders. Regarding RDS, it has been suggested that several gender related factors and hormones have a role in lung development and surfactant production during the intrauterine period [17, 18]. In terms of neurological morbidity, Nuñez et al. [19] discussed the possibility that estradiol, whose values are high in early embryonic development in males, increases the vulnerability of males to neurological disease. Higher cerebral blood flow in male infants was suggested as an optional cause for the difference in this morbidity, as well [20]. Females who were exposed to fetal distress were found to have higher catecholamines in their umbilical cord compare with males. This phenomenon may be indicative of more effective coping by females with fetal distress [21]. In addition, it was suggested that androgens play a role in immunosuppression and hence males are more susceptible to morbidity [22].

Similar to our study, a recent study found no differences in mortality rates between males and females in cases of mixed gender twins [12]. It was suggested that the difference in morbidity and mortality between premature male-male twins and premature female-female twins represents a male disadvantage as opposed to a female advantage and that this disadvantage may be transferred from males to females in unlike-sex twin pairs, perhaps via an intrauterine paracrine effect [10]. Melamed et al. [4] noted that females who are part of a pair of male-female twins had respiratory and neurological complication rates similar to males and higher than that of females who are part of a pair of female-female twins. This finding was also concluded by Steen et al. [11].

Birth order was also studied as a variable. Armson et al. [23] studied the relationship between birth order and neonatal complications among newborns of different gestational ages including <34 weeks of gestational age, a group corresponding to the population in our study. It was found that a second-born, regardless of gender and birth weight, was at increased risk of neonatal complications. In the study of Armson et al. the second twins were less likely to suffer excess morbidity with elective cesarean. This may partially explain the results of our study, since the majority (> 80%) of the newborns were delivered by cesarean section. It should be noted that among twins of different sexes, when the second child was male, more neonatal complications were observed, and when the second twin was female, a similar trend was observed without statistical significance. In a sub-analysis of neonatal complications, among infants born at 34 weeks of gestational age, the second twin had an increased risk of RDS relative to the first twin. Furthermore, it was found that among twins in whom only one had a defined neonatal complication, the risk of second-born complications of intubation, low Apgar scores, mechanical ventilation and RDS was increased relative to the first twin. In addition, the risk of mortality was increased in the second twin when the birth weight was less than 1500 g (similar characteristics to our study population). It should be noted that in this study, as in the current study, the overall analysis revealed no significant differences between the first and second born infants [24].

One of the findings in our study was the inverse association between the week of gestational age at birth and neonatal and postnatal complications among twins of opposite gender. The higher the gestational week at birth, the lower the chance of death and complications, regardless of gender. Similar findings on the relationship between the week of birth and neonatal complications were discussed in a previous study [25]. In our study, a logistic regression model revealed the association between early birth gestational week and low weight Z score as independent risk factors for ROP. This finding is consistent with the study of Lundgren el al [26]., which indicated a similar association among newborns in relation to the gestational week of birth.

The limitations of our study are the retrospective nature and the low number of participants. However, it presents our experience among preterm mixed gender twins and reinforces the assumption of no gender differences in the outcome, when comparing between mixed gender twins.

## Conclusion

Our study suggests that there are no significant differences in neonatal and post neonatal mortality and morbidity among different gender twins with very low birth weight. Our results support the need for further studies.

## Data Availability

The datasets used and/analyzed during the current study are available from the corresponding author on reasonable request.

## Abbreviations

AGA: appropriate for gestational age
BPD: bronchopulmonary dysplasia
CPAP: continuous positive airway pressure
GA: gestational age
IVH: intraventricular hemorrhage
LGA: large for gestational age
LOS: length of stay (hospitalization days)
NEC: necrotizing enterocolitis
PVH: periventricular hemorrhage
PVL: periventricular leukomalacia
RDS: respiratory distress syndrome
ROP: retinopathy of prematurity
SD: standard deviation
SGA: small for gestational age
SRT: surfactant replacement therapy

## References

[1] Mathews TJ, MacDorman MF (2008). Infant mortality statistics from the 2005 period linked birth/infant death data set. Natl Vital Stat Rep.

[2] Zeitlin J, Saurel-Cubizolles MJ, De Mouzon J, Rivera L, Ancel PY, Blondel B, Kaminski M (2002). Fetal sex and preterm birth: are males at greater risk?. Hum Reprod.

[3] Tan H, Wen SW, Walker M, Fung KF, Demissie K, Rhoads GG (2004). The association between fetal sex and preterm birth in twin pregnancies. Obstet Gynecol.

[4] Melamed N, Yogev Y, Glezerman M (2009). Effect of fetal sex on pregnancy outcome in twin pregnancies. Obstet Gynecol.

[5] Stevenson DK, Verter J, Fanaroff AA, Oh W, Ehrenkranz RA, Shankaran S, Donovan EF, Wright LL, Lemons JA, Tyson JE, Korones SB, Bauer CR, Stoll BJ, Papile LA (2000). Sex differences in outcomes of very low birthweight infants: the newborn male disadvantage. Arch Dis Child.

[6] Hoffman EL, Bennett FC (1990). Birth weight less than 800 grams: changing outcomes and influences of gender and gestation number. Pediatrics..

[7] Elsmén E, Hansen Pupp I, Hellström-Westas L (2004). Preterm male infants need more initial respiratory and circulatory support than female infants. Acta Paediatr.

[8] Brothwood M, Wolke D, Gamsu H, Benson J, Cooper D (1986). Prognosis of the very low birthweight baby in relation to gender. Arch Dis Child.

[9] Peacock JL, Marston L, Marlow N, Calvert SA, Greenough A (2012). Neonatal and infant outcome in boys and girls born very prematurely. Pediatr Res.

[10] Shinwell ES, Reichman B, Lerner-Geva L, Boyko V, Blickstein I (2007). ‘Masculinizing’ effect on respiratory morbidity in girls from unlike-sex preterm twins: a possible transchorionic paracrine effect. Pediatrics..

[11] Steen EE, Källén K, Maršál K, Norman M, Hellström-Westas L (2014). Impact of sex on perinatal mortality and morbidity in twins. J Perinat Med.

[12] Ahrenfeldt LJ, Larsen LA, Lindahl-Jacobsen R, Skytthe A, Hjelmborg J, Möller S, Christensen K (2017). Early-life mortality risks in opposite-sex and same-sex twins: a Danish cohort study of the twin testosterone transfer hypothesis. Ann Epidemiol.

[13] Mulla ZD, Plavsic SK, Ortiz M, Nuwayhid BS, Ananth CV (2013). Fetal sex pairing and adverse perinatal outcomes in twin gestations. Ann Epidemiol.

[14] Zhao D, Zou L, Lei X, Zhang Y (2017). Gender differences in infant mortality and neonatal morbidity in mixed-gender twins. Sci Rep.

[15] Horbar JD, Carpenter JH, Badger GJ, Kenny MJ, Soll RF, Morrow KA, Buzas JS (2012). Mortality and neonatal morbidity among infants 501 to 1500 grams from 2000 to 2009. Pediatrics..

[16] Dorling JS, Field DJ, Manktelow B (2005). Neonatal disease severity scoring systems. Arch Dis Child Fetal Neonatal Ed.

[17] Catlin EA, Powell SM, Manganaro TF, Hudson PL, Ragin RC, Epstein J, Donahoe PK (1990). Sex-specific fetal lung development and mullerian inhibiting substance. Am Rev Respir Dis.

[18] McMillan EM, King GM, Adamson IY (1989). Sex hormones influence growth and surfactant production in fetal lung explants. Exp Lung Res.

[19] Nuñez JL, McCarthy MM (2003). Sex differences and hormonal effects in a model of preterm infant brain injury. Ann N Y Acad Sci.

[20] Baenziger O, Jaggi JL, Mueller AC, Morales CG, Lipp HP, Lipp AE, Duc G, Bucher HU (1994). Cerebral blood flow in preterm infants affected by sex, mechanical ventilation, and intrauterine growth. Pediatr Neurol.

[21] Greenough A, Lagercrantz H, Pool J, Dahlin I (1987). Plasma catecholamine levels in preterm infants. Acta Paediatr Scand.

[22] Trigunaite A, Dimo J, Jørgensen TN (2015). Suppressive effects of androgens on the immune system. Cell Immunol.

[23] Armson BA, O'Connell C, Persad V, Joseph KS, Young DC, Baskett TF (2006). Determinants of perinatal mortality and serious neonatal morbidity in the second twin. Obstet Gynecol.

[24] Usta IM, Nassar AH, Awwad JT, Nakad TI, Khalil AM, Karm KS (2002). Comparison of the perinatal morbidity and mortality of the presenting twin and its co-twin. J Perinatol.

[25] Hartley RS, Hitti J (2005). Birth order and delivery interval: analysis of twin pair perinatal outcomes. J Matern Fetal Neonatal Med.

[26] Lundgren P, Kistner A, Andersson EM, Pupp IH, Holmström G, Ley D, Löfqvist C (2014). Low birth weight is a risk factor for severe retinopathy of prematurity depending on gestational age. PLoS One.

